# Mitochondrial Reactive Oxygen Species and Their Contribution in Chronic Kidney Disease Progression Through Oxidative Stress

**DOI:** 10.3389/fphys.2021.627837

**Published:** 2021-04-23

**Authors:** Hasna Tirichen, Hasnaa Yaigoub, Weiwei Xu, Changxin Wu, Rongshan Li, Yafeng Li

**Affiliations:** ^1^School of Life Sciences, Institutes of Biomedical Sciences, Shanxi University, Taiyuan, China; ^2^Shanxi Medical University, Taiyuan, China; ^3^Department of Nephrology, Shanxi Provincial People's Hospital, Taiyuan, China; ^4^Precision Medicine Center, Shanxi Provincial People's Hospital, Taiyuan, China

**Keywords:** reactive oxygen species, mitochondria, oxidative stress, chronic kidney disease, kidney

## Abstract

Mitochondria are known to generate approximately 90% of cellular reactive oxygen species (ROS). The imbalance between mitochondrial reactive oxygen species (mtROS) production and removal due to overproduction of ROS and/or decreased antioxidants defense activity results in oxidative stress (OS), which leads to oxidative damage that affects several cellular components such as lipids, DNA, and proteins. Since the kidney is a highly energetic organ, it is more vulnerable to damage caused by OS and thus its contribution to the development and progression of chronic kidney disease (CKD). This article aims to review the contribution of mtROS and OS to CKD progression and kidney function deterioration.

## Introduction

Chronic kidney disease (CKD) is a major and growing public health burden (Bello et al., 2017). It is defined as an estimated or measured glomerular filtration rate (GFR) <60 ml/min per 1.73 m^2^, or the presence of kidney damage markers, or both, for at least 3 months (Duann and Lin, 2017). It is estimated that approximately 11–13% of the world's population suffers from CKD (Hill et al., 2016). It is often associated with diabetes and hypertension and results in some complications including cardiovascular diseases (CVDs), anemia, kidney disease progression, acute kidney injury, mineral and bone disorders, and cognitive decline (Pendse and Singh, 2005; Jha et al., 2013; Hill et al., 2016).

It is well-established that CKD is a state of elevated oxidative stress (OS). By definition, OS is an imbalance between reactive oxygen species (ROS) production and removal due to overproduction of ROS and/or decreased antioxidant defense activity (Pizzino et al., 2017; Ghosh et al., 2018). The high oxidative activity of mitochondria in the kidney makes the organ prone to the damage caused by OS, which can lead to kidney disease progression and subsequent renal dysfunction (Che et al., 2014; Daenen et al., 2019). Oxidative stress in kidney diseases has been reported to be due to both the depletion of antioxidants and the increased production of ROS (Daenen et al., 2019). A cross-sectional study of CKD patients compared with healthy subjects showed a progressive increase in the levels of the OS marker malondialdehyde (MDA), while concentrations of antioxidant elements, including superoxide dismutase (SOD), glutathione peroxidase (GSH-Px), Zn^2+^, Cu^2+^, and Se^2+^ were lower. The GFR correlated negatively with MDA levels, which resulted in kidney dysfunction (Yilmaz et al., 2006).

It has been demonstrated that an excessive generation of highly ROS is harmful to cells because they result in the oxidation of biological molecules such as lipids, proteins, and DNA (Bae et al., 2011; Daenen et al., 2019). The state of OS in CKD was suggested to be associated with the impaired mitochondrial function and enhanced mitochondrial reactive oxygen species (mtROS) release (Galvan et al., 2017). The oxidant generation process occurs mainly in the mitochondria; the ROS products from this organelle likely contribute to the progression of renal injury and the pathogenesis of atherosclerotic diseases in CKD (Ling and Kuo, 2018). In addition, increased OS is associated with complications such as hypertension, atherosclerosis, inflammation, and anemia in patients at advanced stages of CKD (Daenen et al., 2019).

## Mitochondrial Reactive Oxygen Species Production and Elimination

Reactive oxygen species are produced by various cellular compartments, including the cytoplasm, cell membrane, endoplasmic reticulum, mitochondria, and peroxisome (Forrester et al., 2018). Among these compartments, mitochondria are the major contributor to ROS production; they generate approximately 90% of cellular ROS (Balaban et al., 2005). Superoxide anions are the most abundant ROS in the mitochondria (Zorov et al., 2014). In fact, 0.2–2.0% of the molecular oxygen consumed by mitochondria is reduced to superoxide anions, which are converted to other ROS, such as hydrogen peroxide (H_2_O_2_) and hydroxyl ions (OH^−^) (Duchen, 2004; Balaban et al., 2005).

**Figure 1:**
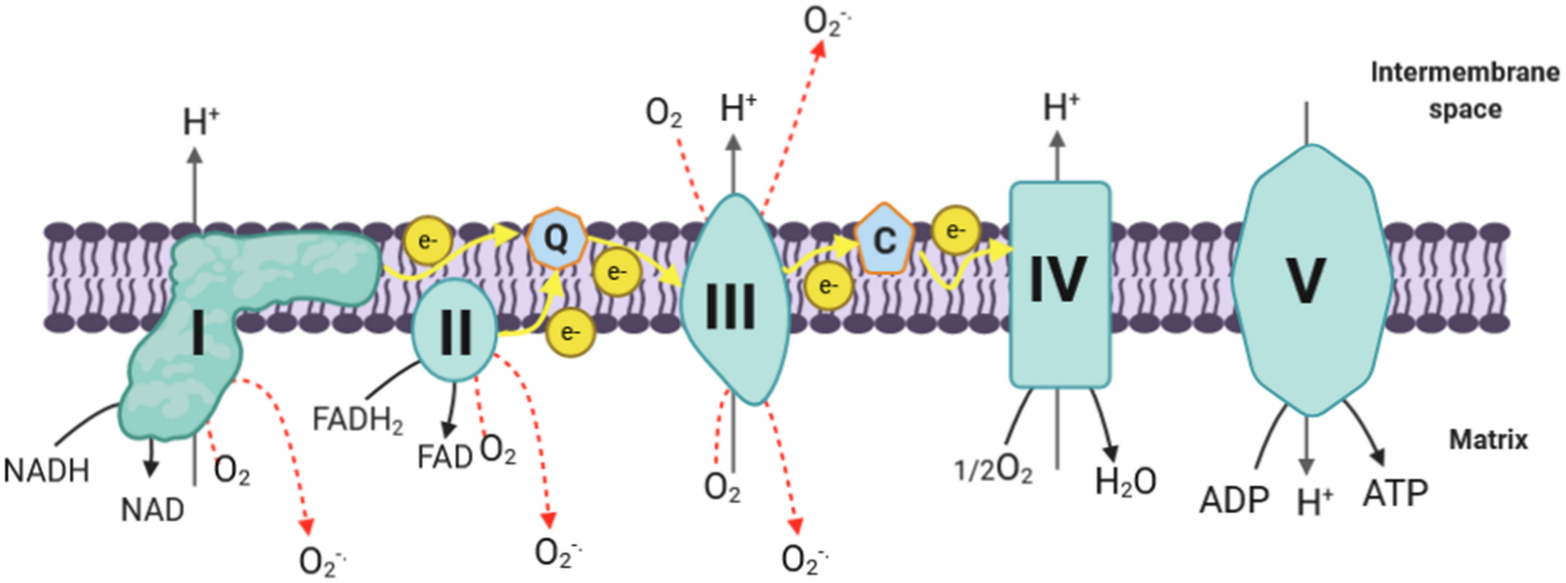
mtROS production in ETC. The electrons donated from NADH in complex I and FADH_2_ in complex II flow to complex III through ubiquinone, and then they are transferred to complex IV via cytochrome *c*; next, the electrons are passed to the molecular oxygen to form water. During this process, complex I, complex III, and complex IV pump protons to the intermembrane space, creating the proton gradient that drives ATP synthesis. During the process of oxidative phosphorylation, electrons leak and interact with molecular oxygen to form superoxide (${\text{O}}_{2}^{-.}$) in complex I and complex III (which are the major ROS production site in the mitochondria) and complex II. Complex III generates superoxide toward the matrix and the intermembrane space, while complex I and complex II produce ROS only toward the matrix. I, complex I; II, complex II; III, complex III; IV, complex IV; V, complex V; Q, coenzyme Q; C, cytochrome *c*; e^−^, electrons; H^+^, protons; ADP, adenosine diphosphate; ATP, adenosine triphosphate; NADH, reduced nicotinamide adenine dinucleotide; NAD^+^, oxidized nicotinamide adenine dinucleotide; FAD, flavin adenine dinucleotide; O_2_, molecular oxygen; ${\text{O}}_{2}^{-.}$, superoxide; H_2_O, water; H_2_O_2_, hydrogen peroxide; SOD, superoxide dismutase; Mn, manganese; Cu, copper; Zn, zinc; mtROS, mitochondrial reactive oxygen species; ETC, electron transport chain.

Mitochondrial reactive oxygen species are mainly produced at the electron transport chain (ETC) during the oxidative phosphorylation process, during which molecular oxygen (O_2_) is reduced to H_2_O (Madamanchi and Runge, 2007; Thomas et al., 2008; Li et al., 2013). In the course of their transport, electrons leak and interact with molecular oxygen to form superoxide (${\text{O}}_{2}^{-.}$) at the flavin mononucleotide (FMN) site of complex I and the Q cycle of complex III, which are the major sources of superoxide and hydrogen peroxide in mitochondria (Hirst et al., 2008; Tahara et al., 2009; Li et al., 2013; Nickel et al., 2014) (Figure 1).

Complex I is composed of a hydrophobic section located in the inner membrane and a hydrophilic section in the matrix, forming an L-shaped structure (Friedrich and Böttcher, 2004; Onukwufor et al., 2019). It transfers two electrons from nicotine adenine dinucleotide (NADH) to ubiquinone (Q) and pumps four protons into the intermembrane space (Ripple et al., 2013). A FMN located at the hydrophilic section of complex I can form FMNH_2_ by accepting the two electrons derived from the oxidation of NADH, which is generated through the tricarboxylic acid cycle (Krebs cycle) in the mitochondrial matrix (Zhao et al., 2019). Electrons are then passed via a series of iron–sulfur clusters arranged from low to high potential to reduce ubiquinone (Q) to ubiquinol (QH_2_) at the Q binding site, which is located at the junction of the membrane arm and matrix arm (Hirst, 2009; Zhao et al., 2019). During this process, mtROS can be generated in the matrix by complex I at both sites of IF (FMN site) and I_Q_ (Q binding site) (Hirst, 2009; Zhao et al., 2019). Additionally, complex I produces mtROS through reverse electron transfer (RET), which is the backward transfer of electrons from QH_2_ to NAD^+^ (Scialò et al., 2017).

Complex III is the major site for ROS generation at the ETC; it has inner (Q_i_) and outer (Q_o_) pools of ubiquinone oriented toward the matrix and the intermembrane space, respectively (Han et al., 2001; Chen et al., 2003). Most of the superoxide, in complex III, is produced as a result of the autooxidation of ubisemiquinone, which is an intermediate produced in complex III during the Q cycle (Trumpower, 1990; Turrens, 2003). It has been demonstrated that ubisemiquinone is the primary direct electron donor capable of reducing O_2_ to superoxide (Turrens et al., 1985; Liu, 1999). During the Q cycle, ubisemiquinone of the Q_o_ site carrying a single electron can move freely in complex III, directly leaking the single electron to O_2_ resulting in ROS generation (Zhao et al., 2019). The Q_i_ site of complex III releases the produced superoxide toward the matrix, while the superoxide generated at the Qo site is released into the intermembrane space (Chen et al., 2003).

Although complex I and complex III are the primary production sites in mitochondria, complex II may produce ROS to a lesser extent. The FAD site of the complex II can produce ${\text{O}}_{2}^{-.}$ toward the matrix. Its production rates by mammalian mitochondria at this complex are very low compared with the rates of superoxide formed at complexes I and II (Lambert and Brand, 2009; Brand, 2010). Complex II generates ROS in both the forward reaction, with electrons supplied by succinate, and the reverse reaction, with electrons supplied from the reduced ubiquinone pool (Quinlan et al., 2012). The rates during succinate oxidation by fumarate reductase, which catalyzes the reverse reaction, are much greater (Quinlan et al., 2012).

Other sites in mitochondria, except the ETC, may produce mtROS. The mitochondrial glycerol-3-phosphate dehydrogenase (mGPDH), which oxidizes the glycerol 3-phosphate and reduces Q to QH_2_ resulting in feeding electrons into the ETC, is capable of generating ROS (predominantly superoxide) in mitochondria (Lambert and Brand, 2009; Brand, 2010; Quinlan et al., 2013). The active site of this enzyme faces the outer side of mitochondrial inner membrane (Lambert and Brand, 2009). Mitochondrial glycerol-3-phosphate dehydrogenase produces superoxide approximately equally toward each side of the mitochondrial inner membrane, suggesting that the Q-binding pocket of mGPDH is the major site of superoxide generation (Orr et al., 2012).

Another site where electrons could escape and form ROS in mitochondria is the electron-transferring flavoprotein–ubiquinone oxidoreductase (ETF-QOR) (Kao et al., 2010). This enzyme is located in the mitochondrial inner membrane and produces superoxide to the matrix during the oxidation of fatty acids where electrons are passed from fatty acids to Q (St-Pierre et al., 2002; Lambert and Brand, 2009; Mailloux et al., 2013b).

The dihydrolipoamide dehydrogenase (Dld) subunit of ketoglutarate dehydrogenase enzyme complex (KGDHC) may be a significant source of mtROS because the flavin of Dld is abundant in mitochondria and has a sufficiently negative redox potential to allow superoxide production (Starkov et al., 2004).

Other sources of mtROS include pyruvate dehydrogenase, 2-oxoglutarate dehydrogenase (Odh), dihydroorotate dehydrogenase, and p66shc/cytochrome *c* (Lambert and Brand, 2009; Mailloux et al., 2013a).

As mentioned above, cellular components other than mitochondria are also capable of producing ROS in the kidney. NADPH oxidases (NOX) are accepted as a major source of ROS generation in diabetic nephropathy and CKD alongside mitochondria (Muñoz et al., 2020). NADPH oxidases have seven different homologs, namely, NOX1–NOX5, DUOX1, and DUOX2, with NOX4 being the most abundantly expressed NOX isoform in the kidney (Irazabal and Torres, 2020). NOX4-derived ROS overproduction in mesangial, endothelial, and tubular cells has been found to be associated with diabetes- and obesity-related kidney disease (Muñoz et al., 2020). Mitochondrial reactive oxygen species production can be increased with the elevation of NOX4 expression, while the downregulation of NOX4 restores mitochondrial bioenergetics and reduces mtROS (Irazabal and Torres, 2020).

**Figure 2:**
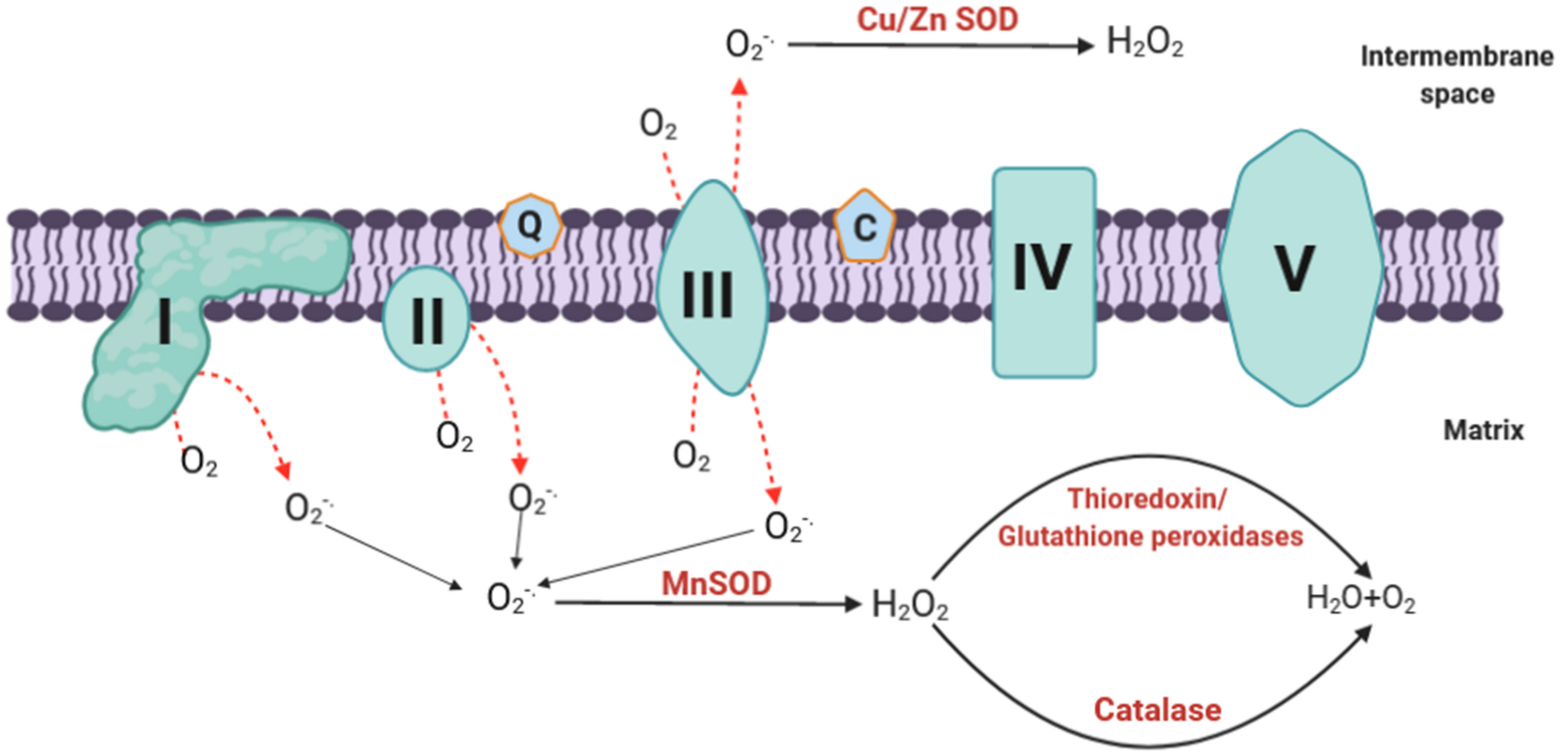
mtROS elimination. The generated superoxide is dismutated to H_2_O_2_ by Mn-SOD at the matrix or Cu/Zn SOD at the intermembrane space. The hydrogen peroxide formed can be degraded by catalase, thioredoxin, and glutathione peroxidases to form water. ${\text{O}}_{2}^{-.}$, superoxide; H_2_O_2_, hydrogen peroxide; SOD, superoxide dismutase; Mn, manganese; Cu, copper; Zn, zinc; H_2_O, water; O_2_, molecular oxygen; mtROS, mitochondrial reactive oxygen species.

To maintain a balanced amount of ROS in the cell, mammalian mitochondria possess various antioxidant enzyme systems that scavenge mtROS as soon as they are generated. The SOD family catalyzes the dismutation of ${\text{O}}_{2}^{-.}$ to H_2_O_2_ by copper and zinc SODs (Cu, Zn-SOD) in the intermembrane space and manganese SOD (Mn-SOD) in the matrix (Fridovich, 1995) (Figure 2). The H_2_O_2_ formed can be toxic; it is therefore degraded following the intervention of specific enzymes including catalase, thioredoxin peroxidase, and GSH-Px (Andreyev et al., 2005; Handy and Loscalzo, 2012) (Figure 2). Catalase is primarily located in peroxisomes; thus, it may not play a central role in eliminating ROS in the mitochondria except in heart and liver mitochondria where it plays an important role in converting H_2_O_2_ to H_2_O (Radi et al., 1991; Rindler et al., 2016). On the other hand, GSH-Px and thioredoxin peroxidase use reduced glutathione (GSH) and thioredoxin (TrxSH) as a substrate, respectively, to reduce H_2_O_2_ to H_2_O. Oxidized glutathione (GSSG) and thioredoxin (TrxS–) are then reduced by glutathione reductase and thioredoxin reductase, respectively, using NADPH as a substrate (Mailloux, 2018). It is noteworthy that the nuclear genome encodes all the antioxidant mitochondrial enzymes, and then they are imported into the mitochondria after being translated into proteins (Li et al., 2013).

Besides enzymatic antioxidants, multiple non-enzymatic antioxidants intervene in the elimination of ROS. These non-enzymatic antioxidants are low-molecular-weight compounds that exist in the plasma, extracellular fluids, intracellular fluids, lipoproteins, and membranes and are categorized as hydrophilic (vitamin C, uric acid, bilirubin, albumin, and flavonoids) or lipophilic (alpha-tocopherol, ubiquinol, and carotenoids) (Ling and Kuo, 2018; Daenen et al., 2019).

## Physiological Role of Mitochondrial Reactive Oxygen Species

Despite taking part in several OS-related pathologies, mtROS are known to have a role in a variety of physiological processes, including proliferation, differentiation, autophagy, immune cell activation, and aging. Under hypoxic conditions, the cell invokes transcriptional and non-transcriptional responses to increase the supply of oxygen while simultaneously reducing oxygen consumption; these adaptations to hypoxia are enhanced by mtROS (Sena and Chandel, 2012; Choi and Kim, 2020). It has been reported that mtROS function as active signaling molecules for diverse cell differentiation, such as stem cell differentiation, myogenic differentiation, and muscle regeneration (Choi and Kim, 2020). In the context of autophagy, mtROS are required to induce autophagy under starvation to promote cell survival or promote controlled autophagic cell death when survival is not possible (Marchi et al., 2012). There is evidence that mtROS play a key role in fighting infections through activation of pathways of adaptive immune and innate immune responses (Sena and Chandel, 2012). It has been suggested that mtROS has a correlation with aging. In fact, mitochondrial dysfunction and consequent excessive ROS production lead to cell senescence and death (Ziegler et al., 2015). However, several studies in *Drosophila melanogaster* and young mice showed that physiologically controlled mtROS might activate adaptive responses that are beneficial to the organism and extend life span (Choi and Kim, 2020).

## Mitochondrial Reactive Oxygen Species and Oxidative Stress in Chronic Kidney Disease

Chronic kidney disease is defined as decreased kidney function shown by a GFR of less than 60 ml/min per 1.73 m^2^, or presence of markers of kidney damage, or both, during at least 3 months, regardless of the underlying causes (Webster et al., 2017). The most common causes of CKD are diabetes and hypertension in all high-income and middle-income countries, and also in many low-income countries (Webster et al., 2017). Chronic kidney disease complications include increased all-cause and cardiovascular mortality, kidney-disease progression, acute kidney injury, cognitive decline, anemia, mineral and bone disorders, and fractures (Jha et al., 2013).

**Figure 3:**
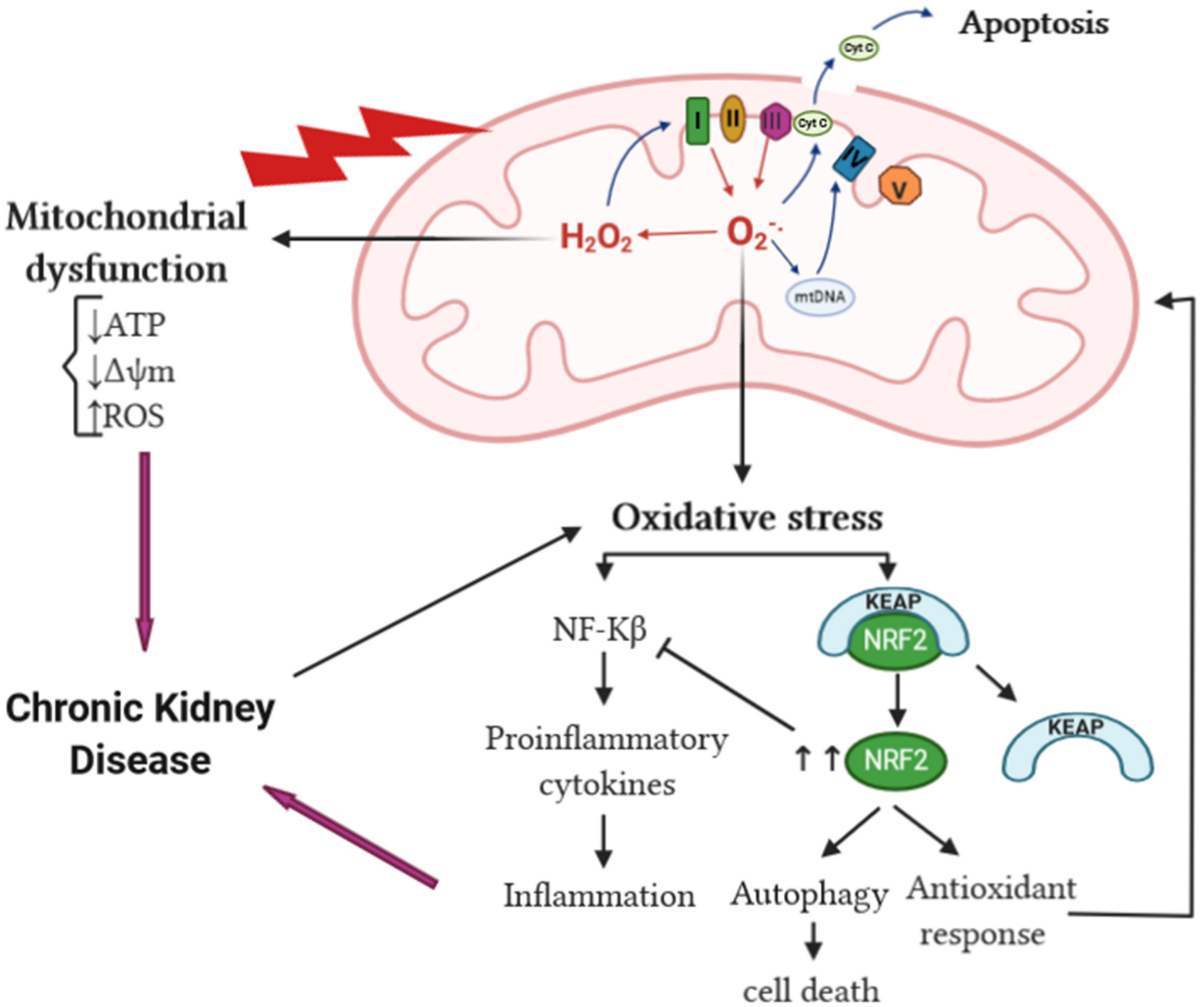
mtROS and OS in CKD. The increase of mtROS can lead to mitochondrial protein, DNA, and membrane damage, in addition to impaired ATP production capacity and lipid oxidation, ultimately leading to mitochondrial dysfunction. Mitochondrial damage can increase the tendency of mitochondria to release cytochrome *c* to the cytosol and thereby activate the cell's apoptosis machinery. The overproduction of superoxide anion can lead to oxidative stress, which in turn activates NF-κB and enhances the inflammatory response resulting in CKD progression. The interaction of Keap1 and Nrf2 is altered by oxidative stress liberating Nrf2 activity from repression by Keap1. In the nucleus, Nrf2 dimerizes with other transcription factors and promotes transcriptional activation of antioxidant and detoxifying enzymes and thereby attenuates the production of proinflammatory cytokines. cyt c, cytochrome *c*; NF-κB, nuclear factor kappa B; Keap1, Kelch-like ECH-associated protein 1; Nrf2, nuclear factor erythroid-derived 2-like 2; mtROS, mitochondrial reactive oxygen species; OS, oxidative stress; CKD, chronic kidney disease.

The kidney requires a significant amount of energy to control the body fluid composition by filtering and reabsorbing materials. The reabsorption is carried out in an ATP-dependent manner; a huge amount of this ATP is supplied by mitochondria (McFarland et al., 2007; Irazabal and Torres, 2020). Therefore, any dysfunction affecting mitochondria will have a crucial impact on renal cellular function. Overproduction of mtROS is linked to mitochondrial dysfunction, which is an early event of kidney injury. Studies have found that mitochondrial dysfunction preceded podocyte fusion and proteinuria in an aldosterone-infused mouse model (Zhu et al., 2011; Su et al., 2013); it can also result in epithelial-to-mesenchymal transition of renal tubular epithelial cells contributing to the fibrogenic process (Granata et al., 2015a). Mitochondrial dysfunction not only precedes kidney injury but also contributes to the development and progression of CKD (Bai et al., 2019), particularly due to a reduction in mitochondrial DNA copy number, loss of mitochondrial membrane potential, and drop of ATP production (Granata et al., 2015a). These changes in mitochondrial function have been linked to mtROS. Hydroxyl radical can damage macromolecules within the mitochondria including DNA (Van Houten et al., 2006); unrepaired mitochondrial DNA damage can lead to complex I and complex III function defect, which results in an increase reduction of O_2_ to form ${\text{O}}_{2}^{-.}$ (Guo et al., 2013). Mitochondrial reactive oxygen species are capable of inducing mitochondrial permeability transition pore, consequently rendering the inner membrane permeable to small molecules (Murphy, 2009). In addition, increased mitochondrial oxidative damage can result in releasing intermembrane proteins to the cytosol such as cytochrome *c* (Murphy, 2009). Mitochondrial impairment can cause a great release of mtROS (Granata et al., 2015a), consequently amplifying OS in CKD, which gives rise to a vicious cycle of excessive mtROS production and mitochondrial dysregulation that eventually triggers cell apoptosis (Guo et al., 2013) (Figure 3).

Studies over the years have implicated ROS and OS in the pathogenesis and development of many diseases including CKD. It has been suggested that OS mediates kidney damage; in CKD and hemodialysis (HD) patients, ROS were increased significantly than in healthy subjects; this increase was associated with high levels of the OS marker 8-hydroxy-2′-deoxyguanosine (8-OHdG) (Granata et al., 2009). In addition, OS is enhanced in CKD patients, especially those with diabetic kidney disease (DKD) (Jha et al., 2016).

Reactive oxygen species can react with other substances and result in different types of OS: superoxide anions react with nitric oxide (NO) to produce peroxynitrite (ONOO^−^), which causes nitrosative stress. The ONOO^−^ formed can oxidize lipids, DNA, and proteins, resulting in modulations of cell signaling and oxidative damage leading to cell necrosis and apoptosis (You and Sharma, 2014). Thus, ONOO^−^ may contribute to the progression of CKD since peroxynitrite, the footprint of ONOO^−^, has been shown to be increased in many human kidney diseases (You and Sharma, 2014). The interaction of H_2_O_2_ and chloride ions (Cl^−^) results in the formation of hypochlorous acid (HOCl), thereby contributing to chlorinated stress (Ling and Kuo, 2018). Hypochlorous acid can induce endothelial dysfunction, decreasing the adhesiveness of extracellular matrix proteins of endothelial cells and converting matrix metalloproteinases into their active form, which leads to the destabilization of vascular and tissue environment surrounding endothelial cells (Ling and Kuo, 2018). In addition, increased formation of advanced glycosylation end products (AGEs) in renal dysfunction can lead to increased carbonyl stress, which can further initiate inflammation in CKD (Ling and Kuo, 2018). Inflammation is the most common outcome of OS in kidney diseases; these two processes are interrelated in a vicious cycle, as each begets and amplifies the other (Tucker et al., 2015). Oxidative stress activates transcription factors such as NF-κB, a redox-sensitive transcription factor that regulates the expression of proinflammatory cytokines and chemokines genes (Cachofeiro et al., 2008) (Figure 3). Furthermore, OS promotes the recruitment and activation of leukocytes, the formation of proinflammatory oxidized lipids, advanced protein oxidation products, and AGEs (Ruiz et al., 2013). Reversely, activated leukocytes, macrophages, and resident cells generate reactive oxygen, chlorine, and nitrogen species, which increases the oxidative state (Ruiz et al., 2013). Mitochondrial reactive oxygen species overproduction contributes to CKD-related chronic microinflammation through the activation of the NLRP3 inflammasome in uremic patients (Granata et al., 2015b). On the other hand, during CKD, the SOD2 is upregulated in response to OS-enhanced nuclear factor erythroid 2-related factor 2 (NRF-2). NRF-2 has been shown by multiple studies to have a renoprotective role in CKD by acting on its downstream SOD2 gene leading to the reduction of ROS in the intracellular environment, therefore promoting cell survival and proliferation. NRF-2 can have an adverse effect in diseases characterized by a proliferative phenotype such as polycystic kidney disease (Zaza et al., 2013; Irazabal and Torres, 2020) (Figure 3).

While ROS and OS cause oxidative damage of different cellular components, autophagy removes oxidized and damaged macromolecules and dysfunctional organelles to restore cellular homeostasis (Kaushal et al., 2019). Autophagy can be implicated in both kidney homeostasis and disease pathogenesis (Ding and Choi, 2015). Existing evidence demonstrates that autophagy has a protective role in the kidney. In renal proximal tubular epithelial cells, mature TGF-β1 protein levels are regulated through autophagic degradation, which suppresses kidney fibrosis induced by unilateral ureteral obstruction (Ding et al., 2014). Inversely, autophagy deficiency in the kidney epithelium of mice was accompanied by elevated ROS production and mitochondrial dysfunction, which were shown to be sufficient to cause many manifestations of FSGS similar to those observed in human idiopathic FSGS kidney biopsy specimens (Kawakami et al., 2015). It has been established that ROS act as an inducer of autophagy. Reactive oxygen species can modify many autophagy-related proteins by direct oxidation of cysteine residues. Cysteine residues located in the α and β subunits of AMPK can be oxidized by ROS leading to AMPK activation and downstream signaling (Kaushal et al., 2019). Oxidation of Atg4 by H_2_O_2_ at the critical Cys-81 residue inactivates the protease, thus facilitating autophagosome formation by Atg8 (Scherz-Shouval et al., 2007). In addition, ROS mediate oxidation of Ask1 resulting in the dissociation of the Bcl-2–beclin-1, making free beclin-1 available for the formation of PI3 kinase/Vps34 complex and subsequent autophagy activation (Kaushal et al., 2019). Oxidative stress also oxidizes mTORC1 and PTEN, which inhibits their activity and promotes autophagy (Kaushal et al., 2019). Under unstressed conditions, Keap1 suppresses Nrf2 activity (Kobayashi et al., 2006). In response to OS, Keap1 is oxidized by ROS-mediated oxidation of cysteine thiols in the cysteine-rich Keap1–Cullin3-based ubiquitin ligase complex, which dissociates Nrf2 from Keap1 and enables Nrf2 to translocate to the nucleus to transactivate target genes, including antioxidant and autophagy genes (Kaushal et al., 2019) (Figure 3). A study, using renal tissue from subtotal nephrectomized CKD model rats, showed impairment in the activation of Nrf2 despite severe OS and inflammation leading to consequent downregulation of the antioxidant enzymes (Kim and Vaziri, 2010). Although it has a renoprotective function against OS, autophagy can lead to cell survival or cell death depending on ROS exposure severity. Mild OS triggers cell survival and repair mechanisms such as the autophagy pathway. Under the conditions of severe OS, ROS levels are excessive for a prolonged period leading to oxidative damage and ultimately cell demise (Sureshbabu et al., 2015).

## Biomarkers of Oxidative Stress in Chronic Kidney Disease

It has been established that OS causes damage to cells, including structural and functional modifications to cellular components, such as membranous lipids, proteins, and DNA. In order to predict the risk of complications in CKD patients, several markers related to OS have been used such as AGEs, oxidized low-density lipoprotein (ox-LDL), MDA, 8-OHdG, and advanced oxidation protein products (AOPPs).

Advanced glycosylation end products are a heterogeneous group of protein and lipids to which sugar residues are covalently bound. Advanced glycosylation end product generation is increased during hyperglycemic conditions such as diabetes mellitus (Bohlender et al., 2005). However, they can also be generated in conditions associated with elevated levels of OS, such as CKD (Stinghen et al., 2016). Advanced glycosylation end products play a role in the high prevalence of endothelial dysfunction and subsequent CVD in CKD patients (Linden et al., 2008). The activation of NOX and mitochondrial pathways by AGEs in both a receptor-dependent manner and a receptor-independent manner leads to an increase in the level of ROS production. In return, high levels of ROS cause an increase of AGEs (Wautier et al., 2001; Lee et al., 2003; Ramasamy et al., 2005).

The enzyme myeloperoxidase (MPO) possesses the ability to convert H_2_O_2_ to various types of ROS including ·OH, ONOO^−^, NO_2_, and HOCl, which in return can modify proteins, lipids, or lipoproteins (Krata et al., 2018). In fact, it has been revealed that in advanced CKD, lipoproteins are more likely to undergo further oxidation to become ox-LDL, whose accumulation stimulates production of proinflammatory cytokines by overlying endothelial cells in patients with kidney diseases (Ozsoy et al., 2006; Krata et al., 2018). Moreover, ox-LDL is found to be highly correlated with atherosclerosis pathogenesis (Ling and Kuo, 2018).

Another marker is the MDA, which is a lipid peroxidation byproduct. It was reported to be increased in CKD, especially in HD patients, and negatively correlated with the GFR (Florens et al., 2016; Ling and Kuo, 2018). Malondialdehyde covalently binds to proteins and nucleic acids, interfering with their normal biological functions (Florens et al., 2016). An elevated serum concentration of MDA is found in uremic patients, and it could be an early prognostic indicator of graft dysfunction after kidney transplantation (Fonseca et al., 2014).

To predict the cardiovascular outcome and mortality in patients with advanced CKD, the asymmetric dimethylarginine (ADMA) has been shown to be a strong and independent predictor (Duni et al., 2017). Asymmetric dimethylarginine is a methylated product of arginine and is the major endogenous inhibitor of nitric oxide synthase (NOS). Also, it was reported that ADMA is negatively correlated with the GFR (De Gennaro Colonna et al., 2009).

8-Hydroxy-2′-deoxyguanosine is one of the biomarkers related to DNA damage by ROS in CKD patients (Ling and Kuo, 2018); it is increased as a result of interaction between oxidative compounds with nucleic acids (Modaresi et al., 2015). This biomarker could be measured in human samples such as in urine samples or even the bloodstream, which makes it a noninvasive systemic OS marker (Pilger and Rüdiger, 2006; Honda et al., 2019). Studies have demonstrated that urinary 8-OHdG reflects an increased systemic level of oxidative DNA damage in DKD patients and severe state of the disease (Xu et al., 2004).

Advanced oxidation protein products are dityrosine-containing byproducts of plasma proteins produced during OS (Selmeci, 2011). Declining renal function was associated with increase in AOPP levels, thus making them good markers of CKD progression (Ling and Kuo, 2018). In fact, it has been shown that plasma levels of AOPPs were at the highest levels in patients on HD, followed by those on peritoneal dialysis and by undialyzed patients with advanced chronic renal failure (Witko-Sarsat et al., 1996).

In order to reduce the possibility of relying on one false-positive or false-negative result, it is recommended to measure a whole panel of biomarkers instead of a single parameter (Gyurászová et al., 2020).

## Pathological Effects of Oxidative Stress in Chronic Kidney Disease

Studies have shown that ROS increase gradually as renal function deteriorates (Terawaki et al., 2004; Dounousi et al., 2006). In fact, OS increases with CKD progression and is inversely and significantly correlated with the level of GFR. Additionally, a study showed that OS presents early during CKD progression (Dounousi et al., 2006). The major consequences of OS in CKD are inflammation and endothelial dysfunction, which induce the progression of different complications such as atherosclerosis, anemia, and amyloidosis (Meenakshi Sundaram et al., 2014; Ling and Kuo, 2018).

### Atherosclerosis

**Figure 4:**
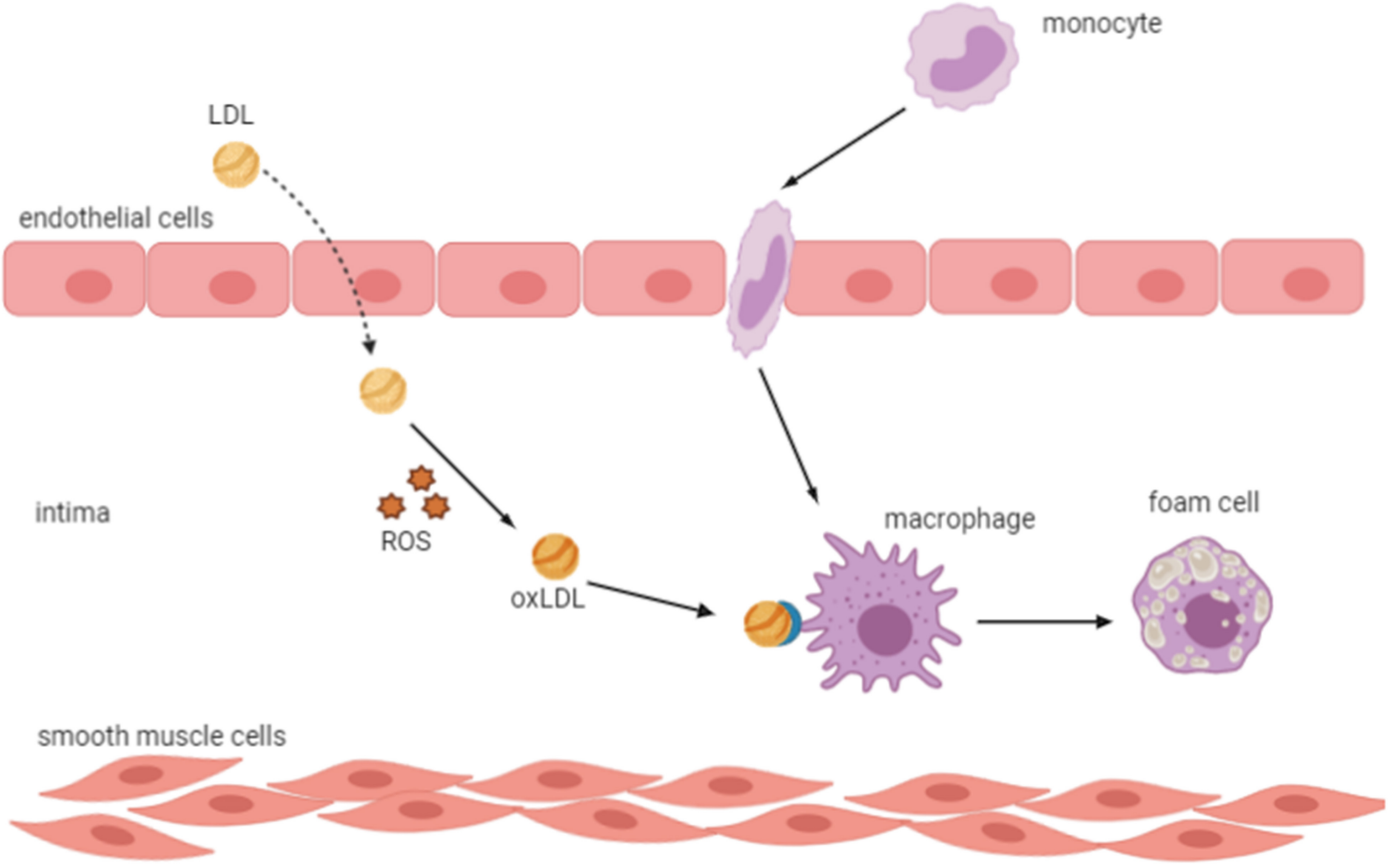
Oxidized LDL transforms macrophages into foam cells. An increase in oxidative stress results in vascular permeability alterations, which allow the entrance of LDL into the vascular intima, where it is oxidized and induces endothelial cells to express leukocyte adhesion molecules and subsequent binding of circulating inflammatory cells and their migration into the subendothelial space. Oxidized LDL is taken up by scavenger receptors of macrophages, which transform into foam cells resulting in the formation of the fatty streak, the initial lesion of atherosclerosis. LDL, low-density lipoprotein; ROS, reactive oxygen species; ox-LDL, oxidized LDL.

In CKD patients, CVD is the major cause of mortality (Ling and Kuo, 2018). In fact, cardiovascular morbidity and mortality increased in all CKD stages even in patients with moderate renal insufficiency (GFR <60 ml/min per 1.73 m^2^) (Cachofeiro et al., 2008). The main cause of CVD is atherosclerosis, a chronic inflammatory disease triggered by the accumulation of cholesterol-containing LDL particles on the arterial wall, which is very prevalent in CKD patients (Cachofeiro et al., 2008; Frostegård, 2013; Bäck and Hansson, 2015). Many studies have shown that OS, chronic inflammation, and endothelial dysfunction are implicated in the development and progression of atherosclerosis (Heitzer et al., 2001; Libby, 2002). Indeed, an increase in the oxidation is directly linked to endothelial dysfunction by reducing the availability of nitric oxide, which resulted from the reaction of ${\text{O}}_{2}^{-.}$ with NO in patients with higher oxidative condition (Münzel et al., 1997; Ling and Kuo, 2018). This results in vascular permeability alterations, thus allowing the entrance of LDL cholesterol into the vascular intima, where it is oxidized and transformed into a highly atherogenic molecule that induces endothelial cells to express leukocyte adhesion molecules and subsequent binding of circulating inflammatory cells as well as their migration into the subendothelial space (Cachofeiro et al., 2008). Oxidized LDL is taken up by scavenger receptors of macrophages, where it is transformed into foam cells resulting in the formation of the fatty streak, which is the initial lesion of atherosclerosis (Libby, 2002; Hansson, 2005) (Figure 4). In response to different growth factors, vascular smooth cells proliferate and migrate to the intima where they produce an extracellular matrix such as collagen and elastin leading to the development of an atheromatous plaque (Stinghen et al., 2016; Kattoor et al., 2017).

Oxidized low-density lipoproteins are important players in the initiation and progression of atherosclerotic changes (Duni et al., 2017). Sources of oxidants include NAD(P)H oxidases, xanthine oxidase, lipoxygenases, mitochondrial respiration, uncoupled NOS, and MPO (Delporte et al., 2013). The MPO enhances the oxidation of LDL by HOCl, which generates a vicious circle effect, prevents resolution of the nascent lesion, triggers OS and lipid accumulation in the subendothelial space, and inhibits normal fibrinolysis (Delporte et al., 2013; Duni et al., 2017). In addition, AGEs may increase the oxidation of LDL by glycation, which results in the development of vascular inflammation due to the formation of antibodies that bind AGEs localized in the vessel wall (Stinghen et al., 2016).

### Amyloidosis

Amyloidosis is a group of complex diseases caused by extracellular deposition of pathological insoluble fibrillary proteins in organs and tissues, and it may result in severe organ dysfunction (Vaxman and Gertz, 2020). The kidneys are one of the most frequent sites of amyloid deposition in both amyloidosis types AL and AA, and several of the hereditary amyloidosis (Dember, 2006).

In OS conditions, ROS contribute to the formation of oxidized amino acids, which directly changes the function of proteins (Meenakshi Sundaram et al., 2014). It has been suggested that protein denaturation due to OS might lead to amyloidosis in long-term HD patients. This is explained by the occurrence of AGEs in β2-microglobulin, the AGEs are formed by the reaction of ROS with other substrates (Meenakshi Sundaram et al., 2014; Ling and Kuo, 2018).

### Anemia

**Figure 5:**
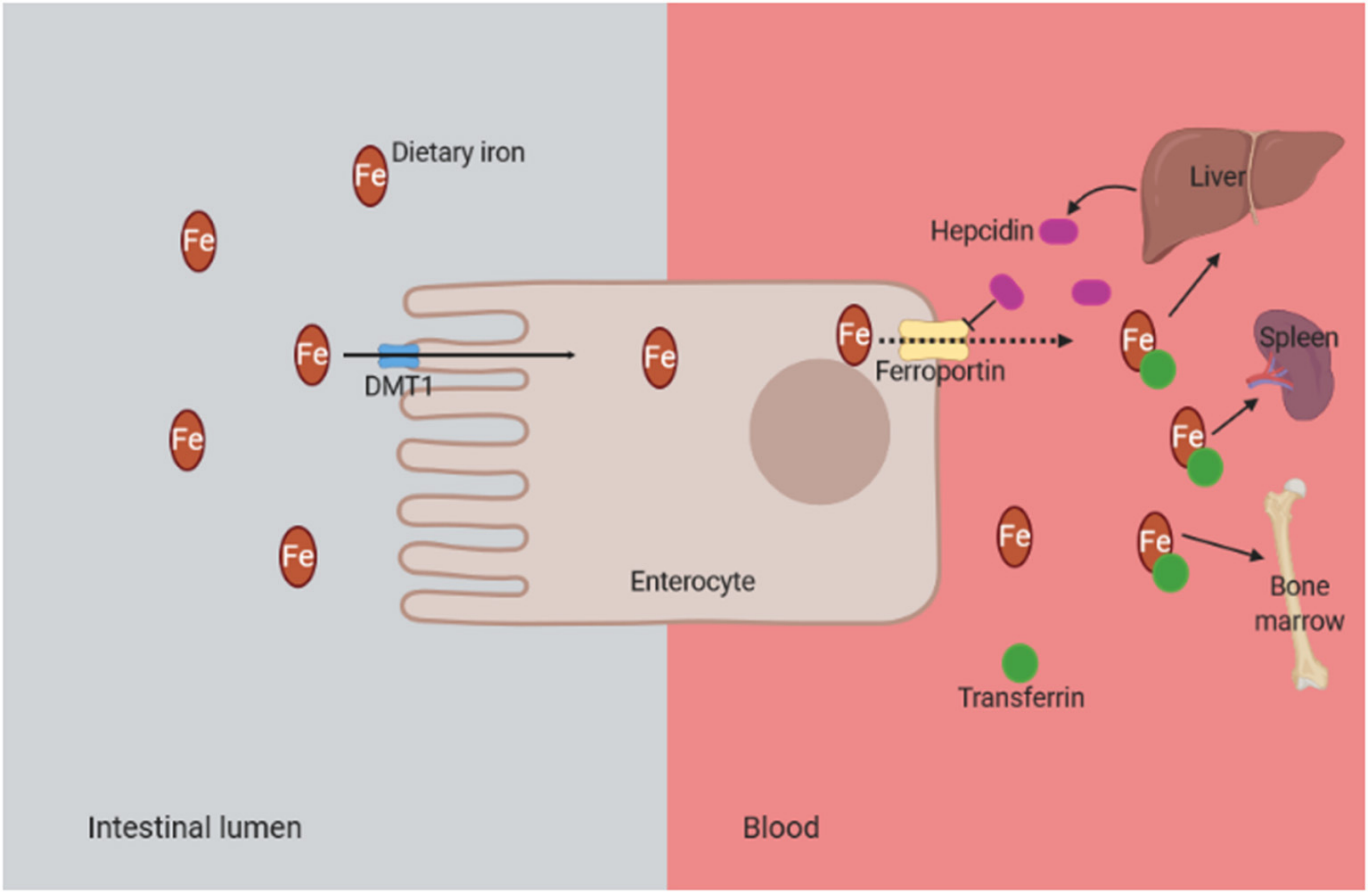
Mechanism of functional iron deficiency anemia in CKD. The presence of chronic inflammation in CKD is associated with overproduction of hepcidin due to inflammatory cytokines such as interleukin-6. Consequently, the hepcidin binds to the iron transporter ferroportin and prevents the dietary iron transport into the circulation. DMT1, divalent metal transporter 1 (it is strongly expressed at the apical brush border of enterocytes, allowing the entry of ferrous iron from the intestinal lumen into cells); CKD, chronic kidney disease.

Anemia is considered as a common complication in CKD patients. Its severity increases as the kidney disease progresses (Smith, 2010). The main cause of anemia development in CKD is the deficiency in erythropoietin production, in addition to inflammation and iron deficiency (Artunc and Risler, 2007; Coyne et al., 2017). In CKD, iron deficiency could be absolute and functional. Absolute iron deficiency anemia is defined as a depletion of iron stores in the bone marrow, liver, and spleen (Gafter-Gvili et al., 2019); and it can be caused by dietary deficiency, low intestinal absorption, and gastrointestinal bleeding (Mehdi and Toto, 2009). On the other hand, functional iron deficiency is defined by normal or increased total body iron stores but with inadequate iron supply for erythropoiesis, and it is mainly associated with inflammation and overproduction of hepcidin (Macdougall et al., 2016). Hepcidin is a hepatic protein, regulated by the inflammatory cytokines such as interleukin-6, that inhibits the absorption of dietary iron in the intestines and impairs the transport of iron from reticuloendothelial system to bone marrow (Mehdi and Toto, 2009) (Figure 5). Consequently, the activation of the inflammation and the overproduction of hepcidin in CKD patients lead to iron deficiency anemia and OS (Nuhu and Bhandari, 2018).

Iron deficiency anemia enhances the susceptibility of eryptosis and increases the risk of OS, which in turn targets the erythrocytes in CKD through increased peroxidation, subsequently leading to modifications in cellular structures and function (Ling and Kuo, 2018; Nuhu and Bhandari, 2018). Consequently, the enhanced susceptibility of erythrocytes to oxidative damage and the risk of ROS production in iron deficiency anemia in CKD lead to a vicious cycle, which results in red blood cell death, anemia, and severity of OS (Nuhu and Bhandari, 2018).

## Conclusion

Overproduction of mitochondrial ROS and the altered antioxidant system play a crucial role in the pathogenesis and the development of CKD through the enhancement of OS in the kidney, thus leading to a dysfunction in cell components and organelles especially mitochondria, resulting in more mtROS production, inflammation persistence, and CKD aggravation. Further investigations of mtROS mechanisms in CKD patients may help in the development of pharmacological strategies targeting mtROS for therapy.

## Author Contributions

HT and HY wrote the paper. CW, RL, WX, and YL revised the paper. HT, HY, and YL designed the outline of the paper. All authors contributed to the article and approved the submitted version.

## Conflict of Interest

The authors declare that the research was conducted in the absence of any commercial or financial relationships that could be construed as a potential conflict of interest.

## Abbreviations

8-OHdG: 8-hydroxy-2′-deoxyguanosine
ADMA: asymmetric dimethylarginine
AGEs: advanced glycosylation end products
AOPPs: advanced oxidation protein products
CKD: chronic kidney disease
Cl^−^: chloride ions
CVD: cardiovascular disease
DKD: diabetic kidney disease
ETC: electron transport chain
FADH_2_: flavin adenine dinucleotide
FMN: flavin mononucleotide
GFR: glomerular filtration rate
GSH: reduced glutathione
GSH-Px: glutathione peroxidase
GSSG: oxidized glutathione
H_2_O_2_: hydrogen peroxide
HD: hemodialysis
HOCl: hypochlorous acid
LDL: low-density lipoprotein
MDA: malondialdehyde
mGPDH: mitochondrial glycerol-3-phosphate dehydrogenase
MPO: myeloperoxidase
mtROS: mitochondrial reactive oxygen species
NADH: nicotine adenine dinucleotide
NADPH: nicotine adenine dinucleotide phosphate
NO: nitric oxide
NOS: nitric oxide synthase
NOX: nicotinamide adenine dinucleotide phosphate oxidase
${\text{O}}_{2}^{-.}$: superoxide
OH^−^: hydroxyl ions
ONOO^−^: peroxynitrite
OS: oxidative stress
ox-LDL: oxidized low-density lipoprotein
Q: ubiquinone
QH2: ubiquinol
RET: reverse electron transfer
ROS: reactive oxygen species
SDH: succinate dehydrogenase
SOD: superoxide dismutase
TrxS^−^: oxidized thioredoxin
TrxSH: reduced thioredoxin.
